# Living Away From Specialized Pollinators: The Pollination System of *Ceiba pentandra* in the Yucatan Peninsula

**DOI:** 10.1002/ece3.70974

**Published:** 2025-02-25

**Authors:** Henry Dzul‐Cauich, Kathryn E. Stoner, Carlos N. Ibarra‐Cerdeña, Miguel A. Munguía‐Rosas

**Affiliations:** ^1^ Departamento de Ecología Humana Centro de Investigación y de Estudios Avanzados del Instituto Politécnico Nacional (Cinvestav) Mérida Mexico; ^2^ School of Natural Resources and the Environment University of Arizona Tucson Arizona USA

**Keywords:** bat‐flower interactions, *Ceiba pentandra*, chiropterophily, pollination system, Yucatan peninsula

## Abstract

Plant‐pollinator systems can exist along the gradient from extreme specialization to extreme generalization. Theoretical work predicts that high pollinator reliability may lead to specialized pollination systems, while pollinator unpredictability may promote generalized pollination systems. However, empirical tests of these predictions are constrained by the availability of accurate field observations, particularly in some groups of plants, such as trees pollinated by nocturnal animals. Plant species that face variable pollinator availability within their distributional range represent an ideal study system to address the effect of pollination predictability on the specialization level of pollination systems. This is the case of *Ceiba pentandra* , a tropical tree with chiropterophilic flowers. Most previous studies have described the pollination system of this tree species as mainly or exclusively bat‐pollinated. Here we studied the pollination system of *C. pentandra* in the Yucatan Peninsula, a region where no species of specialized nectarivorous bats occur. We assessed quantity (visitation rate) and quality pollination components (pollen deposition, fruit/seed set) for diurnal and nocturnal visitors in two different locations and years. We expected to find a generalized pollination system due to the absence of specialized pollinators. As predicted, we found five functional groups of nocturnal and diurnal pollinators. Diurnal and nocturnal pollinators contributed to a similar extent to quantity (visitation rate) and quality pollination components (fruit & seed set). However, the contribution of diurnal and nocturnal pollinators also varied either spatially or temporally. We conclude that the pollination system of *C. pentandra* in the Yucatan Peninsula is highly generalized and that temporal and/or spatial unpredictability in the effectiveness of its pollinators may explain this finding.

## Graphical abstract

The pollination system of *C. pentandra* was addressed. This system was highly generalized.

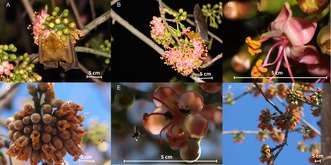

## 1 Introduction

Pollinators have long been recognized as major drivers of floral phenotype and species divergence in angiosperms (Darwin 1862; Stebbins 1970; Harder and Johnson 2009). This fact is best illustrated by the convergence of a suite of floral traits in unrelated plant species that rely on the same pollinator type for sexual reproduction, known as pollination syndrome (Faegri and Pijl 1979; Fenster et al. 2004; Rosas‐Guerrero et al. 2014). Although the original concept of pollination syndrome had the implicit idea that plant‐pollinator systems tend toward greater specialization (Stebbins 1970; Faegri and Pijl 1979), accumulated field observations today have revealed that pollination systems can exist along the gradient from extreme specialization to extreme generalization (Waser et al. 1996; Johnson and Steiner 2000).

Theoretical models predict that specialization to a particular pollinator (or to a functional group of pollinators) evolves when fitness gained exceeds the losses of becoming less adapted to other pollinators, or in other words, when there exist strong pollinator trade‐offs (Aigner 2001; Muchala 2007). Conversely, if pollinator trade‐offs are weak or null, generalized pollination systems are favored (Aigner 2001). An ecological condition that seems to be a reliable predictor for the level of specialization of pollination systems is the degree of predictability of the main pollinator(s) (Waser et al. 1996; Johnson and Steiner 2000). That is, specialized pollination systems are more likely to evolve when effective pollinators are spatially and temporally predictable while the opposite (unpredictable pollinators) would lead to generalized pollination systems (Waser et al. 1996; Aigner 2001). For example, chiropterophilous columnar cacti are highly specialized for bat pollination in the tropics where resident populations of some bat species occur, while highly generalized pollination systems (i.e., pollinated by bats, birds, and insects) can be found in extratropical cacti where fewer species of migratory bats exist (Valiente‐Banuet et al. 1996; Fleming et al. 2001; Freilij et al. 2023). In turn, generalized pollination systems may also facilitate the colonization of new habitats with a depauperate pollinator fauna or where specialized pollinators are either scarce or highly variable (Martén‐Rodríguez et al. 2010; Kriebel and Zumbado 2014). In addition to its effect on pollination systems, pollinator unreliability may also promote autonomous or mixed breeding systems that ensure reproduction when facing pollinator scarcity (Lobo et al. 2005; Fenster and Marten‐Rodriguez 2007). For instance, Martén‐Rodríguez et al. (2010) found a high incidence of autonomous self‐pollination in ornithophilous Gesnerieae in the Antillean islands.

Transitions in the specialization level of the pollination systems are common in some plant lineages (e.g., Tripp and Manos 2008; Brito et al. 2017). Although some of these transitions are mediated by changes in floral traits with high developmental complexity (e.g., bilateral flower asymmetry, corolla fusion and large corolla length; Tripp and Manos 2008; Barret et al., Barrett 2013), minor variations in floral biology may also mediate major transitions in the pollination system. For example, geographic variation in the pollination system from highly specialized (exclusively bat pollinated) to generalized (bats, birds, and insects contribute to pollination) is found in some columnar cacti, and this pattern is explained by variations in only a few hours (3–5 h) in nectar production and flower longevity (Banuet 2002; Fleming et al. 2001; Munguía‐Rosas et al. 2009). Minor changes in floral rewards (from pollen to nectar) have also underlined the transition from specialization to generalization in pollination systems in buzz‐pollinated Miconidae species (Brito et al. 2016; Brito et al. 2017).

Although predictions exist regarding what factors may shape the degree of specialization of pollination systems, the ability to test these is constrained by the deficit of detailed field observations of plant‐pollinator systems. In fact, the level of specialization of plant‐pollinator interactions is unknown for a large proportion of plant species (Johnson and Steiner 2000; Martén‐Rodríguez et al. 2010). This lack of basic information is uneven across plant groups and is particularly scarce for trees (Rosas‐Guerrero et al. 2014) and nocturnally pollinated plants (McGregor and Scott‐Brown, Macgregor and Scott‐Brown 2020). Although the identity of plant visitors can easily be documented, this information alone may artificially increase the level of generalization in pollination systems because some visitors may not be effective pollinators (Waser et al. 1996; King et al. 2013). For instance, Muchala (2006) observed that hummingbirds and bats visited 10 species of *Burmeistera* in Ecuador. However, hummingbirds effectively transferred conspecific pollen for only one of the 10 species studied. Another study showed that although several diurnal animals visited the flowers of the tree *Ceiba pentandra* and touched the reproductive organs, only pollen deposited at night was able to penetrate the ovules (Gribel et al. 1999). Therefore, for an accurate assessment of the level of specialization in pollination systems, quantity (i.e., visiting rate) and quality (pollen deposition, pollen germination and fruit/seed set) components of the pollination process must be documented (e.g., Herrera 1989; Ne'eman et al. 2010).

Although the chiropterophilic pollination syndrome in general predicts more accurately the main pollinators than other syndromes, this widely varies among plant groups (Rosas‐Guerrero et al. 2014). While for some groups of plants (e.g., *Ruellia*) the bat‐pollination syndrome seems to be evolutionarily irreversible (Tripp and Manos 2008), in other groups such as chiropterophilous columnar cacti, there exists latitudinal variation in the specialization level of the pollination system even within the same species (e.g., *Pachycereus pecten‐aboriginum* ; Valiente‐Banuet et al. 2004). Based on these observations, no clear pattern has been found between pollinator availability and pollination systems of chiropterophilous plants. That is, less reliable specialized pollinator availability does not necessarily indicate the plant will possess a more generalized pollination syndrome. However, this relationship has not been evaluated in tropical trees.

*Ceiba pentandra* (Malvaceae) is a chiropterophilous tree with a wide pantropical distribution (Dick et al. 2007). Although several diurnal (birds, nonflaying mammals, insects) and nocturnal animals (bats, hawkmoths and marsupials) visit its flowers, diurnal visitors are usually discarded as effective pollinators owing to their visiting behavior (e.g., Toledo 1977; Janson et al. 1981; Gribel et al. 1999). Diurnal visitors either do not touch the stigma or the stigma is not receptive during the day (Gribel et al. 1999; Quesada et al. 2004; Singaravelan and Marimuthu 2004). Based on this information, the pollination system of *C. pentandra* has largely been considered mainly or exclusively bat‐pollinated (Elmqvist et al. 1992; Gribel et al. 1999; Quesada et al. 2004). However, to our knowledge, quality pollinator components of diurnal vs. nocturnal visitors have not been evaluated in this tree species. Also, the abundance and specialization level of Phyllostomid bats are highly variable across the distribution of *C. pentandra* (González‐Gutiérrez et al. 2022). For example, in the tropical dry forest of the Pacific Coast of Mexico, this tree is visited and exclusively pollinated by specialized nectarivorous bats (mainly *Leptonycteris yerbabuenae* ; Quesada et al. 2004). In contrast, few or no bats at all visited the flowers of some trees of this species in a humid forest in the Osa Peninsula of Costa Rica (Lobo et al. 2005). Thus, *C. pentandra* is an excellent model to assess the effect of variation in pollinator availability on the pollination system of chiropterophilous plant species.

Here we assessed the pollination system of *C. pentandra* in the Yucatan Peninsula, where no nectar‐specialized bat species occur (Arita and Santos del Prado 1999). Instead, a frugivorous bat (*Artibeus jamaicencis*) seems to be an opportunistic pollinator of this tree species in the region (MacSwiney et al. 2017; Dzul‐Cauich and Munguía‐Rosas 2022). Frugivorous bats are thought to be less effective as pollinators compared to highly specialized nectarivorous bats (Heithaus 1982; Fleming et al. 2009). For instance, specialized nectarivorous bats ( *Eonycteris spelaea* ) exhibited a greater visitation rate and carried more pollen than primarily frugivorous bats (*Rousettus leschenaultia*) that visited six night‐blooming plant species—including *C. pentandra* —in southern Thailand (Stewart and Dudash, 2017). However, no observations of potential diurnal pollinators have been conducted in this region. Therefore, we assessed quantity (visiting rate) and quality pollination components (pollen deposition, fruit & seed set as well as seed germination) for nocturnal and diurnal visitors of *C. pentandra* . To assess the predictability of pollinators and their effectiveness, we measured these components in two different sites and flowering seasons. Our main research questions were: Which are the main pollinators of *C. pentandra* ? Is its pollination system generalized? How reliable (in space and time) are pollinators in terms of their effectiveness? Since no specialized nectarivorous bats occur in the study area, we predict a generalized pollination system where nocturnal and diurnal visitors participate, but their relative effectiveness will be variable in space and/or time.

## 2 Materials and Methods

### 2.1 Study System

*Ceiba pentandra* is an emergent tree with a widespread pantropical distribution (Dick et al. 2007). In Mexico, this tree species is deciduous and inhabits seasonally dry and evergreen tropical forests from Sinaloa to Chiapas on the Pacific coast and from Veracruz to Yucatán on the Atlantic coast (Penington and Sarukhán, Pennington and Sarukhán 2005). Fruits are dry capsules, and seeds are dispersed mainly by wind (Penington and Sarukhán, Pennington and Sarukhán 2005). Flowering is massive (tens of thousands of flowers are produced for ca. 4 weeks; Gribel et al. 1999) and starts by the end (or early beginning) of the year in the northern hemisphere (Lobo et al. 2003). Flowers typically open at night and are arranged in inflorescences; corollas are pale pink, consisting of 5 petals, five stamens, and an exerted stigma (Gribel et al. 1999; Penington and Sarukhán, Pennington and Sarukhán 2005). The flowers of *C. pentandra* produce abundant nectar as the main reward (up to 10 L per tree per night and up to 200 L per flowering season; Gribel et al. 1999). Flowers and their rewards attract a variety of visitors; however, often only bats (Phyllostomid bats in the Neotropics and Pteropodid bats in the Paleotropics) are recognized as the main or the exclusive effective pollinators (Baker and Harris 1959; Gribel et al. 1999; Singaravelan and Marimuthu 2004). *Ceiba pentandra* can be self‐compatible or self‐incompatible, but flower visitors often lead to greater reproductive success (Murawski and Hamrick 1992; Lobo et al. 2005).

The study was conducted at two localities approximately 300 km apart in the Yucatan Peninsula. The first belongs to the municipality of Merida in the state of Yucatan (21°2′3.68 “N, 89°45′52.21” W; 10–14 m a.s.l. Hereafter only Merida) and the second to the municipality of Benito Juarez in the state of Quintana Roo (21°1′47.43″ N, 87°7′3.42″ W; 8–10 m a.s.l. Hereafter only Cancun). Both sites were found in tropical dry forest with moderate human disturbance. The most characteristic woody species present in the region are: *Lysiloma latisiliquum* (Fabaceae), *Gymnopodium floribundum* (Polygonaceae), *Acacia milleriana* (Fabaceae), *Mimosa bahamensis* (Fabaceae), *Diospyros anisandra* (Ebenaceae) and *Piscidia piscipula* (Fabaceae) (Islebe et al. 2015). The weather is tropical subhumid with summer rains with precipitation varying from 600 to 800 mm/year (Islebe et al. 2015). The predominant soils are lithosol (Islebe et al. 2015).

### 2.2 Flower Visitors

The study was conducted at the two study sites for two consecutive reproductive seasons: December–March 2022–2023 and December–March 2023–2024 (hereafter, for simplicity, named only by the year each season began: 2022 or 2023). Since *C. pentandra* may reach up to 40 m tall in the study area, only trees with accessible branches and flowers (i.e., trees 8–25 m tall with branches 4–5 m from the forest floor) were used for the study. From this group, a sample of 100 trees (at least 4 km apart) was randomly chosen: 50 from Merida and 50 from Cancun. However, sample size varied slightly among different components of the study due to logistical limitations (see details below).

To identify nocturnal flower visitors, one inflorescence (with 10–34 flowers) per tree was video recorded with a Sony Handy Cam (DCR‐SR85, Beijing, China) using the night shot function. An independent infrared lamp was used as a supplementary source of light to improve the quality of the images (IRLamp6, Wildlife Engineering, Pennsylvania, USA). Video recordings started with anthesis and lasted for 2 h. This procedure has been successfully performed in other populations of *C. pentandra* in the same region (Dzul‐Cauich and Munguía‐Rosas 2022). Video recording with infrared light does not disturb the activity of nocturnal visitors, and the period of filming typically coincided with peak foraging of Phyllostomid bats (Stoner et al. 2002; Lobo et al. 2005). The same camera was used for filming diurnal visitors but using natural light. Diurnal filming started at sunrise (ca. 0600 h) and lasted for the same period as for nocturnal visitors. The same trees filmed during the night were filmed the following morning; however, a different tree was filmed each time. Video recording was simultaneously performed in Merida and Cancun with the help of a group of field assistants trained by the first author. In total, 93 inflorescences from distinct trees (43 in Merida and 50 in Cancun) were filmed during the 2 years the study lasted. All videos were examined in slow motion to identify the visitors to the finest taxonomic level. The number of visits, as well as their feeding behavior (i.e., if visitors fed on pollen or nectar and if they contacted the reproductive organs of the flowers) was documented.

We captured bats flying around flowering trees using three mist nets (9 × 12 m) for 3 h (1900–2100) around 10 reproductive trees per site (a different tree each night; five nights in 2022 and five nights in 2023). All samplings were conducted during the flowering peak (mid‐January) and full moon nights were avoided. Each bat caught was identified in situ to the species level using specialized, local keys (Medellín et al. 2007). Hematophagous and specialized insectivore bats were excluded from data analyses. A sample of pollen on the fur (face and chest) of captured bats was taken using a brush and then placed on microscope slides (Dafni 1992). Once in the laboratory, the slides were examined under a microscope (magnification: 40×) to identify pollen of *C. pentandra* by comparison with a reference collection. Samples from bats with more than 10 grains of *C. pentandra* were considered flower visitors and potential pollinators of the study species. All bats caught were released unharmed after species identification and pollen collection. Bat sampling was conducted with the authorization of local laws (Permit: SPARN/DGVS/04285/22). The species captured are not at risk or under the protection of local or international laws.

### 2.3 Floral Biology and Flower Rewards

During 2023, 40 flowers from two different trees per site (*n* = 80 flowers, 4 trees) were observed during the whole night until 1200 the following day. For each flower observed, time to anthesis, pollen dehiscence, and stigma receptivity were recorded. Stigma receptivity was determined every 2 h by their appearance (turgor, color, etc.) as well as stigma response after adding a few drops of H_2_O_2_ (visible bubbles appear on a receptive stigma; Dafni 1992). The time to flower wilting and corolla abscission was also recorded. For the same number of flowers from the same inflorescence and trees, nectar volume and concentration were measured every hour from anthesis until nectar production stopped. Nectar volume was measured with a commercial hypodermic syringe, and nectar concentration was measured with a portable hand refractometer (Hanna instruments, HI96832, Washington D.C, USA).

### 2.4 Pollination Components for Diurnal and Nocturnal Visitors

#### 2.4.1 Visitation Rate

Visitation rates (visits·inflorescence·h) of effective visitors (flower visitors that touched reproductive organs) during the night and the day were calculated from the video recordings mentioned in the subsection *Flower Visitors*.

#### 2.4.2 Pollen Load Size

Ten to 34 flowers per tree were assigned to one of the following four exclusion treatments (five inflorescences per tree, *n* = 93 trees; 43 in Merida and 50 in Cancun): (i) Nocturnal pollination: flowers were exposed only to nocturnal visitors and covered with mesh bags from sunrise (ca. 0600 h) to the dehiscence of the corollas. (ii) Diurnal pollination: flowers were covered at the mature bud stage with mesh bags all night and uncovered the following morning (from 0600 to corolla dehiscence) to be exposed only to diurnal visitors. (iii) Open pollination: flowers were exposed to all visitors (diurnal and nocturnal). (iv) all visitors excluded: flowers were excluded during their whole lifetime. Treatments iii and iv represented positive and negative control groups, respectively. Flowers allocated to these treatments were not emasculated because pollen is an important reward, and its removal may artificially reduce pollinator attraction.

A random sample of three flowers per inflorescence per treatment (*n* = 65 flowers per treatment) was collected and fixed in FAA (formaldehyde, acetic acid and ethanol) and taken to the laboratory. The flowers were dissected to extract the styles and then softened in 1 N KOH at 65°C for 20 min, rinsed with distilled water, and stained for 20 min at 65°C in aniline blue (Dafni 1992). Finally, the stained styles were mounted individually on glass slides. For each style, the number of conspecific pollen grains on the stigma was counted under a fluorescence microscope (Leica DM2500LED, Wetzlar, Germany).

#### 2.4.3 Fruit and Seed Set

We tagged a random sample of seven flowers per inflorescence allocated to each one of the four treatments described above (see the *Pollen load size* subsection) (*n* = 2604 flowers from 372 inflorescences, 172 in Merida and 200 in Cancun). Tagged flowers were checked once a week until the flower either aborted or set fruit; then we used the proportion of fruit produced in the sample to calculate fruit set. To assess seed set, we collected another group of 5–10 mature fruits per tree (*n* = 342 fruits; 143 in Merida [96 in 2022 and 47 in 2023]; 199 in Cancun [118 in 2022 and 81 in 2023]) and opened them to count seeds. Additionally, we counted the ovules in four flowers from four different trees per site and used the average (314 ovules) as the denominator of seed number to calculate seed set (i.e., seeds/ovules).

#### 2.4.4 Seed Germination

From the fruits collected as described in the subsection *Fruit and seed set*, 50–100 apparently viable seeds per fruit were taken and then all seeds from each treatment/site/year combination were pooled. A sample of 50 seeds from each pool was randomly selected. Seeds were scarified with fine sandpaper (180–200 grit) and disinfected with a 1.5% solution of hypochlorite. Since persistent fungal infection is common in seeds of this tree species (Dzul‐Cauich and Munguía‐Rosas, Unpublished Observation), the seeds were also sprayed with a fungicide (quaternary of ammonium compounds and glutaraldehyde; ANIBAC Cítrico, Promotora Técnico Industrial, Jiutepec, Mexico). Groups of five seeds from each treatment combination were placed in Petri dishes (*n* = 800 seeds in 160 dishes, 10 replicates per treatment combination) with a disc of filter paper at the bottom. Seeds were kept in a growth chamber (Binder Inc., KBWF 720, Tuttlingen, Germany) at a constant temperature of 26°C and a photoperiod of 12 h light and 12 h dark. Light was provided by high‐pressure sodium lamps (PAR = 56.59 μmol m^2^ s^−1^). Seed germination (radicle emergence) was recorded every 48 h for 30 days.

### 2.5 Statistical Analyses

Flower visitors observed in the videos were separated into five functional groups. Three groups of nocturnal visitors: big bats (*Artibeus* spp.), small bats ( *Glossophaga mutica* ) and nocturnal insects (owlet moths [Noctuidae], and an unidentified species of net‐winged insect [Neuroptera]), and two groups of diurnal visitors: hummingbirds (Trochilidae) and bees (Apidae). To assess if the frequency distribution within these functional groups was spatially and temporally variable, the frequency distribution was compared between sites and years with a goodness of fit test using a chi‐square test. Pollination components were fitted to mixed‐effects generalized linear models with the exclusion treatment, the site, and the year as the main sources of variation. Since the issue of main interest was to assess how the pollination components varied in space and time, only those interactions containing the exclusion treatment (exclusion × site, exclusion × year, and exclusion × site × year) were included in the models. In all models, the individual tree was included as a random factor to account for repeated measures or the lack of independence among flowers in the same tree. A Poisson error distribution was used for models with pollination visitation rate and pollen load size as response variables. For fruit set, seed set, and seed germination, a binomial error distribution was utilized. In the case of pollinator visitation rate, the pollinator exclusion treatment only had two levels (diurnal and nocturnal) because filming the controls made no sense (negative controls were excluded from visitors at all times) and was logistically challenging (positive controls were exposed to visitors 16–17 h).

When significant differences among the levels of pollination exclusion treatments were found, post hoc Tukey's tests were conducted to identify significant differences between pairs of levels (except for visiting rate which only have two levels). When an interaction was significant, the nature of each interaction was examined visually with interaction plots, and we also conducted a partitioned analysis of the least squared means. Since only the interaction with diurnal and nocturnal levels were of interest, mean square's partitioning was only conducted for these levels.

All the analyses were performed using R 4.0.3 (R Core Team 2020). All raw data are available as Data S1.

## 3 Results

### 3.1 Flower Visitors

**FIGURE 1:**
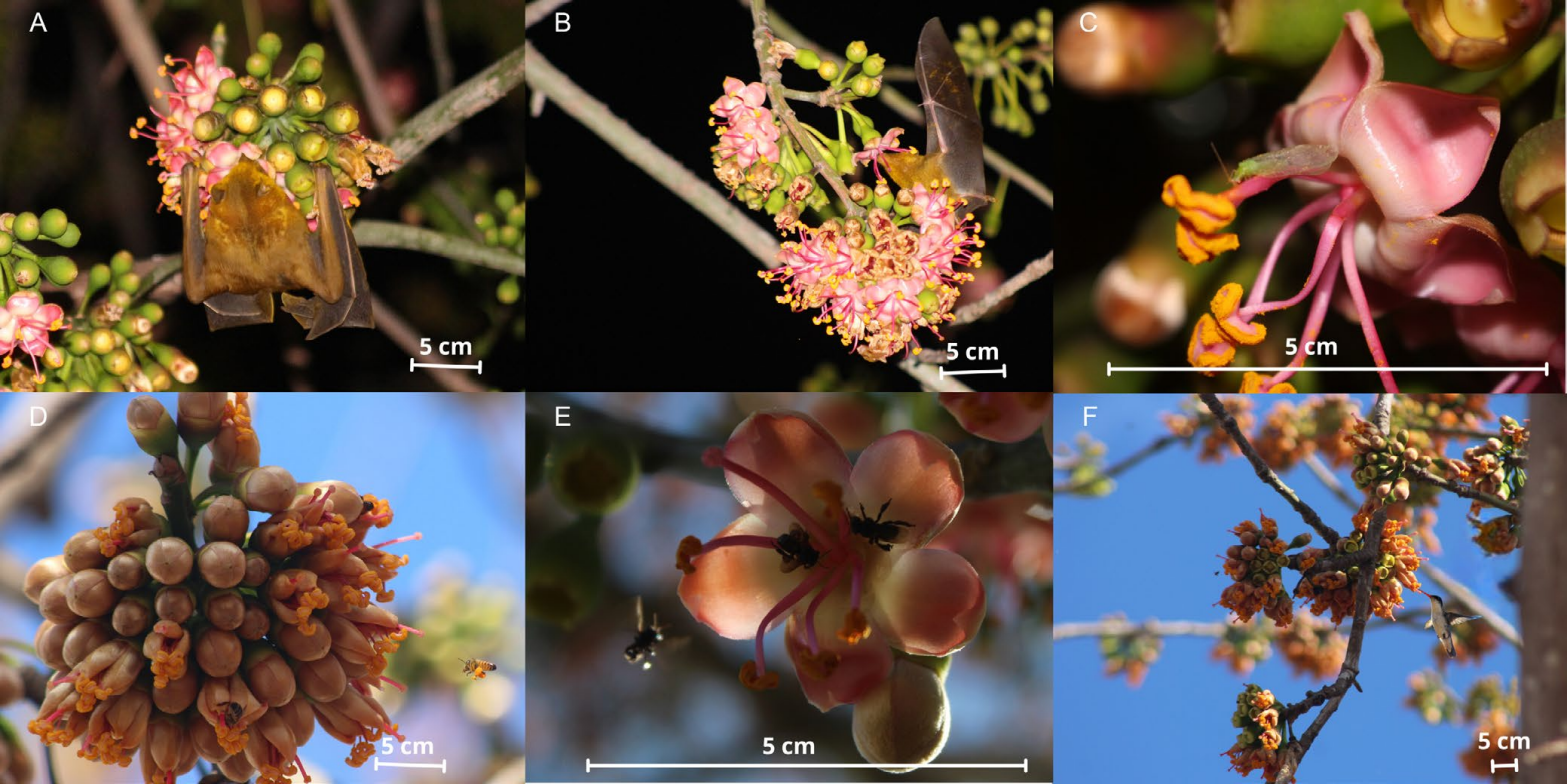
Flower visitors of *Ceiba pentandra* : *Artibeus jamaiciencis* (A), *Glossophaga mutica* (B), Neuroptera (C), Apidae bees (D, E), Trochilidae hummingbird (F). All photos were taken with either white light (A, B, C) or natural light (D, E, F). Scale bars represent 5 cm in all cases.

Two frugivorous (*A. jamaiceiencis* and *A. lituratus* ) and one omnivorous ( *Glossophaga mutica* ) bat species, as well as some nocturnal insects (Owlet moths and net‐winged insects), were recorded visiting the flowers at night (Figure 1A–C). Using mist nets, 308 bats with pollen of *C. pentandra* on their fur were captured. From these, 96.11% were *A. jamaiciencis* and 2.60% were *A. lituratus*. Only one individual of *G. mutica* and one of *Sturnira parvidens* with pollen of *C. pentandra* were captured at only one site (Cancun). Therefore, together these last two bat species represented only 0.65% of captures. In contrast, daytime recordings documented only two Apidae bee species and one Trochilidae hummingbird species visiting the flowers (Figure 1D–F). All of the flower visitors mentioned above touched the reproductive organs of the flowers and therefore are potential effective pollinators of *C. pentandra* .

According to our recordings, nocturnal visitors accounted for 43.40% to 66.34% of visits, while diurnal visitors accounted for 33.66% to 56.60% of visits (Table 1). For both study sites and during the two years of the study, the main nocturnal visitors were *A. jamaiciencis* and the main diurnal visitors were *Apidae* bees. However, there were statistically significant differences between sites (χ^2^_4_ = 10.14, *p* = 0.04) and years (χ^2^_4_ = 15.57, *p* < 0.01) in the observed frequency of the groups of flower visitors (Table 1).

**TABLE 1:** Percentage of flower visitors of the tree *Ceiba pentandra* recorded at two sites in the Yucatan Peninsula (Merida & Cancun) and for two reproductive seasons (years: 2022 and 2023). The subtotals indicate the percentage grouped by visitor guild: Nocturnal vs. diurnal. The frequency of visits (n) is also shown in brackets.

		Nocturnal				Diurnal		
Site	Year	Bat 1	Bat 2	Insects	Subtotals	Bees	Hummingbirds	Subtotals
Merida	2022	33.67 (33)	4.08 (4)	27.55 (27)	65.30 (64)	26.53 (26)	8.17 (8)	34.70 (34)
	2023	24.53 (13)	7.55 (4)	11.32 (6)	43.40 (23)	56.60 (30)	0 (0)	56.60 (30)
Cancun	2022	36.47 (31)	8.23 (7)	16.47 (14)	61.17 (52)	34.10 (29)	4.73 (4)	38.82 (33)
	2023	46.15 (48)	10.58 (11)	9.61 (10)	66.34 (69)	30.78 (32)	2.88 (3)	33.66 (35)

Bat 1 = frugivore, *Artibeus spp*.

Bat 2 = Omnivore, *Glossophaga mutica* .

### 3.2 Floral Biology and Flower Rewards

Full anthesis of all examined flowers occurred in the evening from 1900 to 1930 h and remained open until midday (1130–1230 h) of the following day. Stigmas showed symptoms of receptivity (bright, turgent and positive reaction to H_2_O_2_) immediately after anthesis, and pollen dehiscence occurred 30–60 min after anthesis. Early visual symptoms of wilting appeared in the morning (ca. 1000 h). However, corolla dehiscence did not occur before 1100 h. In fact, some pollen grains were available on most of the examined anthers as late as 1100–1200 h.

**FIGURE 2:**
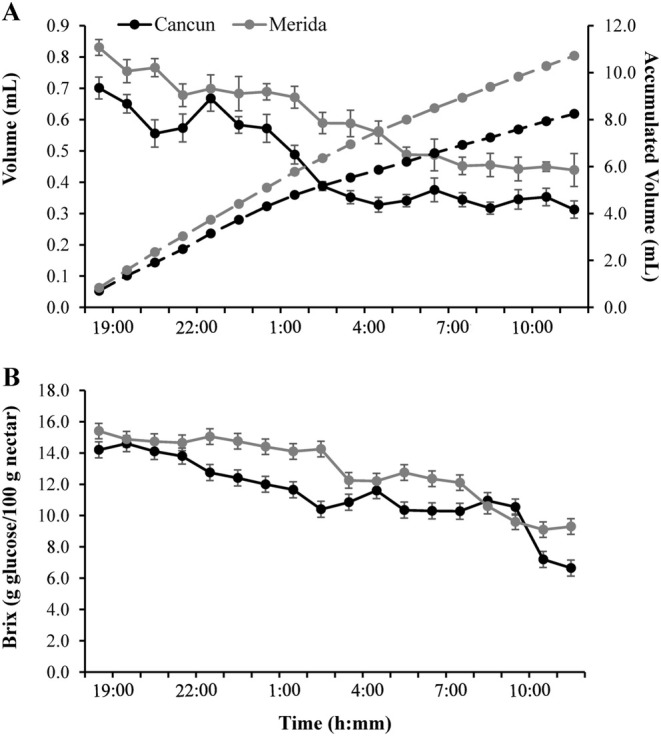
Nectar volume (A) and nectar concentration (B) of the flowers of the tree *Ceiba pentandra* in two localities (Merida & Cancun) in the Yucatan Peninsula. In A, nectar volume produced each hour (continuous lines) and the volume of nectar accumulated (dotted lines) are shown. Data in all cases are mean values ±1SE, except for accumulated volumes.

Regardless of the study site, nectar chambers were full of nectar by the time of anthesis (ca. 1900 h). Nectar production slowly and steadily dropped through the night (1900–0400 h; Figure 2A). However, flowers continued producing relatively small quantities of nectar (0.3–0.5 mL) during the first 6 h of the day (0600–1100 h; Figure 2A). Therefore, accumulated nectar production reached maximum values near midday (1000–1200 h; Figure 2A). Nectar concentration showed its maximum values (14–16 Brix) immediately after anthesis and dropped slowly and steadily during the night and the morning of the following day, reaching its lowest values (8–10 Brix) from 1000 to 1200 h (Figure 2B). The temporal pattern depicted for nectar concentration was virtually identical at both study sites (Figure 2B).

### 3.3 Pollination Components for Diurnal and Nocturnal Visitors

#### 3.3.1 Visitation Rate

**FIGURE 3:**
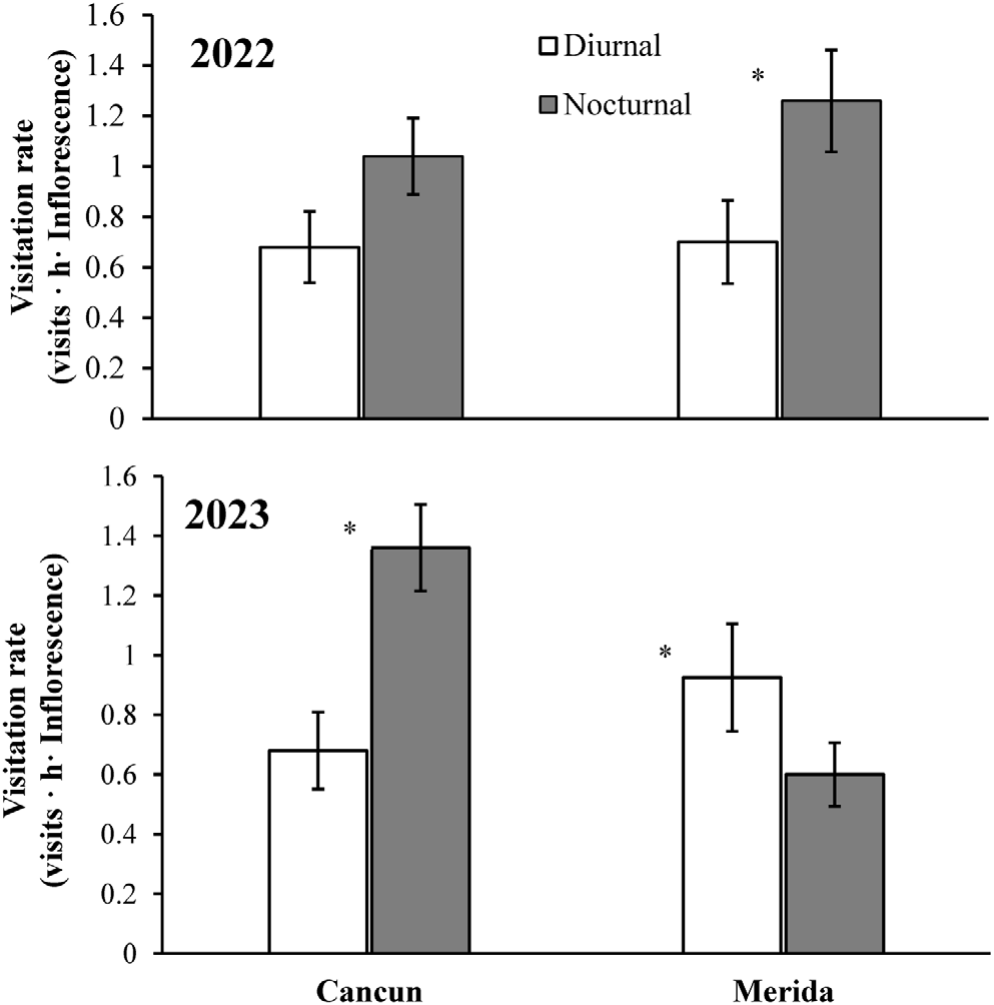
Flower visiting rate of diurnal and nocturnal pollinators of the tree *Ceiba pentandra* in two sites in the Yucatan Peninsula (Merida & Cancun) observed for two different years (2022, 2023). Data are mean values ±1 SE. The asterisk indicates statistically significant differences between diurnal and nocturnal pollinators for each site × year combination.

In general, diurnal and nocturnal visitors exhibited a similar visitation rate (Tables 2 and 3). Although this rate was not different between years and sites, the triple interaction (pollinator exclusion × site × year) was statistically significant (Table 2). That is, the relative importance of diurnal and nocturnal visitors was spatially and temporally variable. The visitation rate of nocturnal visitors was greater than that of diurnal visitors in Merida in 2022 and in Cancun in 2023 (Figure 3). However, the opposite pattern (diurnal visitors outperformed nocturnal visitors) was recorded in Merida in 2023, and no difference between diurnal and nocturnal visitors was observed in Cancun in 2022 (Figure 3).

**TABLE 2:** Results of statistical analyses to assess the effect of pollinator exclusion treatment (Exclusion), site (Merida & Cancun) and year (2022 & 2023) and all the interactions with pollinator exclusion treatment on five pollination components (pollinator visits [Visits], pollen load, pollen germination on the stigma, fruit set, seed set and seed germination) of the tropical tree *Ceiba pentandra* in a tropical dry forest in the Yucatan Peninsula. All the values are Type II Wald chi‐square tests. The subscripts indicate the degrees of freedom.

	Pollination component				
Source of variation	Visits	Pollen load	Fruit set	Seed set	Seed germination
Exclusion	*Χ*_*1*_^2^ = 0.03	*Χ*_*3*_^2^ = 6282**	*Χ*_*3*_^2^ = 17.78**	*Χ*_*3*_^2^ = 69.49**	*Χ*_*1*_^2^ = 1.57
Site	*Χ*_*1*_^2^ = 0.07	*Χ*_*1*_^2^ = 7.21**	*Χ*_*1*_^2^ = 3.39	*Χ*_*1*_^2^ = 1.64	*Χ*_*1*_^2^ = 1.06
Year	*Χ*_*1*_^2^ = 0.46	*Χ*_*1*_^2^ = 12.05**	*Χ*_*1*_^2^ = 39.27**	*Χ*_*1*_^2^ = 16.52**	*Χ*_*1*_^2^ = 32.88**
Exclusion × Site	*Χ*_*1*_^2^ = 0.01	*Χ*_*3*_^2^ = 15.39**	*Χ*_*3*_^2^ = 5.14	*Χ*_*3*_^2^ = 0.43	*Χ*_*1*_^2^ = 1.33
Exclusion × Year	*Χ*_*1*_^2^ = 0.14	*Χ*_*3*_^2^ = 218.06**	*Χ*_*3*_^2^ = 0.44	*Χ*_*3*_^2^ = 2.74	*Χ*_*1*_^2^ = 6.81**
Exclusion × Site × Year	*Χ*_*1*_^2^ = 4.05*	*Χ*_*3*_^2^ = 1.05	*Χ*_*3*_^2^ = 0.06	*Χ*_*3*_^2^ = 1.33	*Χ*_*1*_^2^ = 0.01

*p < 0.05; **p < 0.01.

**TABLE 3:** Pollination components (Component) indicating the effectiveness of nocturnal and diurnal flower visitors of the tree *Ceiba pentandra* in the Yucatán Peninsula. Open pollinated flowers (Open) and bagged flowers (excluded to all visitors: All excluded) were included as positive and negative control groups, respectively. All data are mean values ±1 SE. Different superscript letters indicate statistically different pairs of means based on post hoc multiple comparisons.

Component (Units)	Nocturnal	Diurnal	Open	All excluded
Visits (visits/h/inflorescence)	0.94 ± 0.16^a^	1.02 ± 0.19^a^		
Pollen load (grains/stigma)	397.66 ± 8.14^a^	351.69 ± 7.71^b^	487.88 ± 8.71^c^	170.34 ± 4.62^d^
Pollen germination (%)	4.81 ± 0.17^a^	3.48 ± 0.15^b^	4.09 ± 0.12^a,c^	3.66 ± 0.21^b,c^
Fruit set (%)	25.96 ± 1.94^a^	23.35 ± 1.99^a^	27.57 ± 2.79^a^	5.98 ± 2.08^b^
Seed set (%)	79.79 ± 1.85^a^	81.68 ± 1.86^a^	64.75 ± 5.46^a^	37.70 ± 4.92^b^
Seed germination (%)	34.00 ± 6.17^a^	23.50 ± 5.31^a^	21.00 ± 7.00^a^	34.00 ± 6.00^a^

#### 3.3.2 Pollen Load Size

Although the pollen load size of flowers exposed to only diurnal and only nocturnal visitors differed statistically, pollen loads attributable to nocturnal visitors were only 1.13 times bigger than those of flowers only exposed to diurnal visitors (Table 3). Flowers exposed to all visitors received significantly more pollen grains than either diurnal (1.39 times more pollen) or nocturnal visitors alone (1.23 times more pollen). The flowers excluded from all visitors received a far lower quantity of pollen grains (Table 3).

**FIGURE 4:**
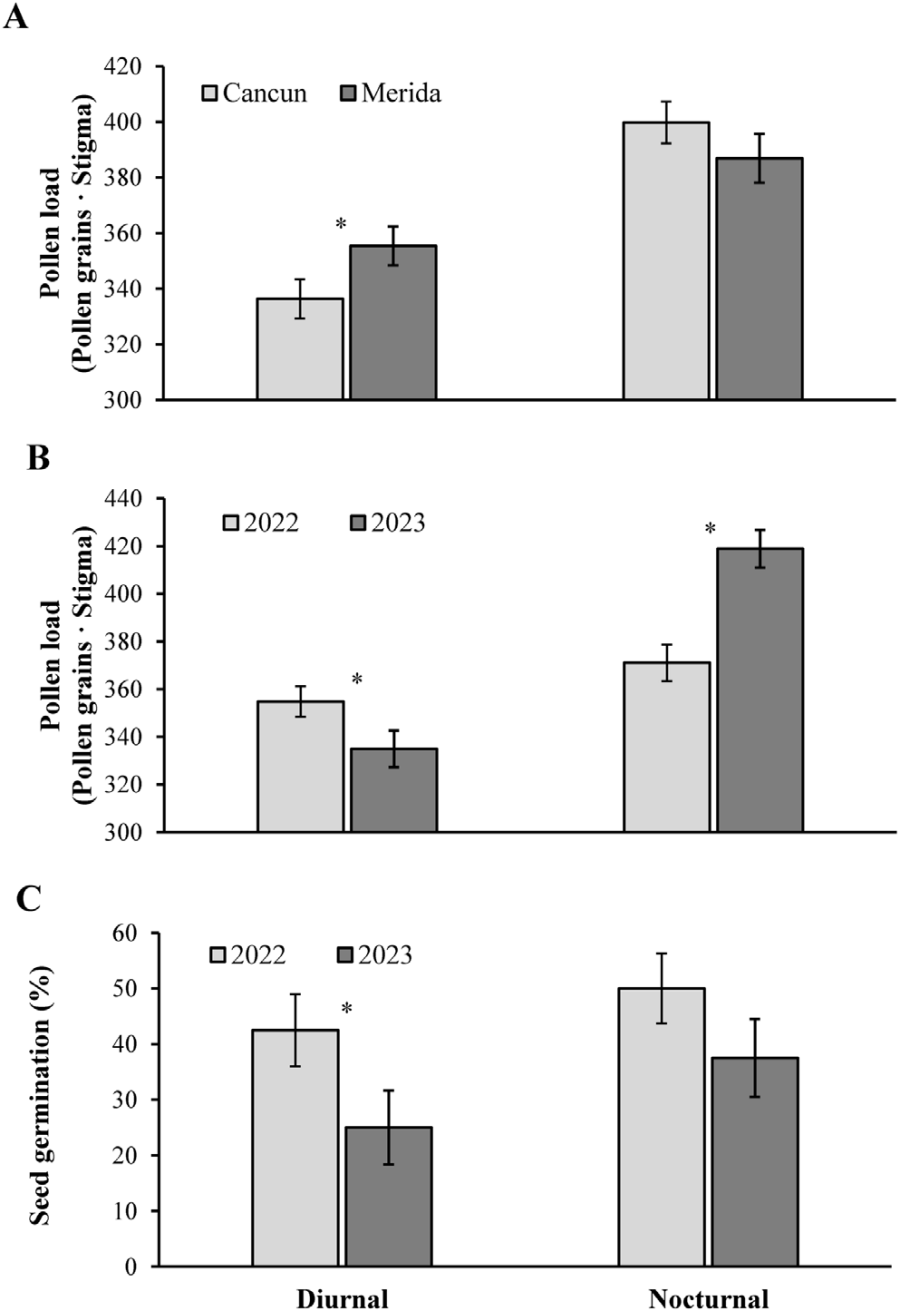
Pollen load sized on the stigma (A & B) and seed germination (C) of the tree *Ceiba pentandra* at two sites (Merida & Cancun; A) and years (2022 & 2023; B & C). Data are mean values ±1 SE. The asterisk indicates statistically significant differences between pairs of means.

Pollen load size also was significantly variable across sites (Cancun: 352.74 ± 9.51 grains·stigma; Merida: 369.53 ± 10.40 grains·stigma) and years (2022: 371.00 ± 6.53 grains·stigma; 2023: 354.03 ± 10.43 grains·stigma) (Table 2). Moreover, the interactions pollinator exclusion × site and pollinator exclusion × year were statistically significant for this variable (Table 2). Differences in terms of pollen load size between sites were detected for diurnal but not for nocturnal visitors (Figure 4A). Finally, pollen loads attributed to diurnal visitors were greater in 2022, while those attributed to nocturnal visitors were greater in 2023 (Figure 4B).

#### 3.3.3 Fruit and Seed Set

Fruit and seed set were significantly affected by the pollination exclusion treatment (Table 2). Fruit and seed set of flowers exposed to either diurnal or nocturnal pollinators were similar to the values observed in flowers exposed to all pollinators; however, these three groups (diurnal, nocturnal and open pollinated flowers) exhibited significantly greater fruit and seed set than flowers excluded from all visitors (Table 3). Finally, fruit and seed set were both significantly variable between years (Table 2). Fruit set and seed set were 1.87 and 1.21 times greater in 2022 (fruit set = 31.42% ± 1.86%; seed set = 86.71% ± 9.40%) than in 2023 (fruit set = 16.77% ± 1.80%; seed set = 71.43% ± 2.56).

#### 3.3.4 Seed Germination

Among the main sources of variation examined, significant differences were only found between years (Table 2). In 2023, seed germination 30 days after sowing (42.50% ± 6.53%) was 2.83 times greater than in 2022 (15.00% ± 3.91%). However, the interaction of pollinator exclusion treatment × year was also significant (Table 2). That is, significant differences between years were found in flowers only exposed to diurnal visitors but not for those flowers exposed to nocturnal visitors (Figure 4C).

## 4 Discussion

In contrast to previous studies reporting a mainly or exclusively bat‐pollinated system of *Ceiba pentandra* in areas with a rich community of specialized species of nectarivorous bats (Elmqvist et al. 1992; Gribel et al. 1999; Quesada et al. 2004), our study shows that, in an area where specialized nectarivorous bats are absent (the Yucatan Peninsula), nocturnal (frugivorous & omnivorous bats and insects) and diurnal (bees and hummingbirds) animals visited the flowers of *C. pentandra* (quantitative component of pollination) at similar rates. In addition, diurnal and nocturnal visitors deposited substantial conspecific pollen grains, which led to similar fruit and seed set (qualitative components of pollination). This suggests that the pollination system of *C. pentandra* is highly generalized, and thus, this tree species maximizes its pollination success with the whole community of flower visitors (nocturnal and diurnal). We suggest that the absence of specialized nectarivorous bats and the observed spatial and temporal variation of pollinator effectiveness may explain the generalization found in the pollination system of *C. pentandra* in the Yucatan Peninsula, as predicted by theory (Waser et al. 1996; Aigner 2001).

Although the morphology of the flowers of *C. pentandra* in the Yucatan Peninsula largely coincided with the chiropterophily pollination syndrome, the extended flower anthesis, stigma receptivity, and nectar production allowed the participation of different functional groups of diurnal visitors (bees and birds) during the pollination process of this tree species. Similarly, variation in the pollination system from highly specialized (exclusively bat‐pollinated) to generalized (bats, birds and insects contributed to pollination) observed in chiropterophilous columnar cacti was explained by variation in a few hours (3–5 h) in nectar production and flower longevity (Banuet 2002; Fleming et al. 2001; Munguía‐Rosas et al. 2009). Although geographic variation in the specialization level of pollination systems has been reported in other species of chiropterophilous plants (e.g., Columnar cacti; Fleming et al. 2001; Banuet 2002), cases of intraspecific geographic variation in plants with this pollination syndrome are rare in the literature (but see Valiente‐Banuet et al. 2004). The large floral display, attractive to effective pollinators even within its nonnative range of distribution, as well as the shift from specialization to generalization in the pollination system may have allowed *C. pentandra* to successfully colonize new habitats, and this, in turn, may partially explain its large distribution across the globe (Dick et al. 2007). Future detailed studies of *C. pentandra* across its distribution range will help test this proposed explanation.

The Yucatan Peninsula is a particular region of Mesoamerica in terms of the composition of the community of nectarivorous bats (see Figure 4 in Arita and Santos del Prado 1999). Only one species of glossophagine bat occurs ( *G. mutica* ) in this region (Arita and Santos del Prado 1999), and this species is documented as omnivorous, feeding on nectar, fruit, and insects as a source of protein (Herrera et al. 2001; Clare et al. 2014). In contrast, 7 to 9 specialized nectarivorous bat species are found in other regions of this part of the continent (Valiente‐Banuet et al. 1996; Arita and Santos del Prado 1999; González‐Gutiérrez et al. 2022). Although previous reports of *A. jamaicencis* visiting the flowers of *C. pentandra* in the Yucatan Peninsula exist (MacSwiney et al. 2017; Dzul‐Cauich and Munguía‐Rosas 2022), this interaction can be considered as unique since *A. jamaicencis* is not among the top five bat species involved in bat‐flower interactions in Central and North America ( *L. yerbabuenae* , *G. mutica* , *G. morenoi* & *C. mexicana* ; González‐Gutiérrez et al. 2022). Despite being locally abundant in the tropical dry forest of Jalisco, Mexico, *A. jamaicencis* did not visit flowers of *C. pentandra* , where the specialized nectarivore *L. yerbabuenae* was the main pollinator (Quesada et al. 2004). Therefore, we suggest that *A. jamaicencis* is an opportunistic pollinator of *C. pentandra* in the Yucatan Peninsula.

Some traits exhibited by specialized nectarivorous bats of the New World (elongated rostrum/tongue, reduced incisors/M and the ability to exploit nectar while hovering) have made them highly efficient pollinators (Heithaus 1982; Fleming et al. 2009). In contrast, frugivorous bats have shorter rostrums and well‐developed molars/incisors that allow them to efficiently harvest and consume fruit, but these traits can make nectar exploitation harder and often may result in destructive flower visits (Heithaus 1982; Fleming et al. 2009; Stewart and Dudash, 2017). We suggest that the relative suboptimal pollinator service provided by frugivorous bats such as *A. jamaicencis*, together with the observed spatial and temporal variation in their effectiveness as pollinators of *C. pentandra* (see significant interactions in Table 2) may have been a selective pressure that favored the unusually long flower longevity, stigma receptivity, and nectar production observed in *C. pentandra* in the Yucatan Peninsula. These conditions likely favored an extended stigma receptivity and nectar production, allowing the participation of diurnal pollinators, which increased the fitness of this tree species. While the production of floral rewards ceased as early as 0230–0300 h in Samoa (Elmqvist et al. 1992) and Brazil (Gribel et al. 1999), in our study area, nectar production lasted until mid‐day of the following day. In other words, nectar production in the Yucatan lasted more than two times longer than that reported by some previous studies. This strategy would be energetically profitable only if it increases the pollination success (Ashman and Schoen 1996; Pyke and Ren 2023). As mentioned before, *A. jamaicencis* appears to be an opportunistic flower visitor of *C. pentandra* in the Yucatan, since its activity as a pollinator depends on the availability and attractiveness of other resources (i.e., fruit) available simultaneously in the region (MacSwiney et al. 2017). Under this uncertainty, a temporal extension in the production of floral rewards, although costly, should be profitable.

Interestingly, despite the chiropterophilous syndrome exhibited by the flowers of *C. pentandra* , we found ca. 27% of fruit set attributable to diurnal visitors. Therefore, we suggest that diurnal pollinators are not mere secondary pollinators (i.e., less‐effective or less‐frequent floral visitors that occasionally contribute to pollination; Rosas‐Guerrero et al. 2014; Jaeger et al. 2023) because they appear to contribute at the same rate to reproductive success as do nocturnal visitors (Table 3). This fact dramatically contrasts with previous studies of this species. For example, in the Amazonia, it was shown that diurnally pollinated flowers of *C. pentandra* did not set fruit or seed at all (Gribel et al. 1999). The fact that both diurnal and nocturnal visitors are involved in the pollination of *C. pentandra* and that fruit and seed set attributed to either only diurnal or only nocturnal visitors were not significantly different from that observed in open‐pollinated flowers (Table 3) suggests that there exists a high redundance between these two guilds of visitors. High levels of redundance between diurnal and nocturnal pollinators are expected to evolve when the main pollinators are unreliable in space and/or time to ensure sexual reproduction (Fleming et al. 2001).

Diurnal insects have been considered poor pollinators of *C. pentandra* because they do not touch both reproductive organs and because they typically do not move among different trees (e.g., Munguía‐Rosas 2003; Nathan et al. 2005). Although the visiting behavior of some birds suggests that they may pollinate *C. pentandra* (Toledo 1977), this fact has not been experimentally tested. Despite their small size, bees often touched anthers and the stigma while exploiting pollen and nectar available during the day. Although these insects move mainly within the same tree, this may promote geitonogamous crosses and set viable seeds in self‐compatible plant species. This may be the case in the study populations since a self‐compatible breeding system has been reported in this tree species (Lobo et al. 2005; Murawski and Hamrick 1992) and we observed up to 6% of fruit set when all pollinators were excluded in the study trees (Table 3). It is likely that this percentage of fruit set was mainly due to geitonogamous crosses since the flowers were not emasculated and the entire inflorescence was covered with the same mesh bag. Some pollen from different flowers might be involuntarily deposited on the stigmas during experimental manipulations.

Our experimental design also allowed us to assess spatial and temporal variation in the quality and quantity of pollination components of diurnal and nocturnal pollinators in the study area. The significant third‐order interaction observed for pollinator visits (a quantity component) and the two second‐order significant interactions for pollen load size (a quality component) strongly suggest significant spatial and temporal variation in different components of nocturnal and diurnal pollination (Table 2). That is, the relative importance of diurnal and nocturnal pollinators changes in time and/or space. These findings conform with theoretical predictions and our own expectations that spatial and temporal uncertainty in pollinator service may be associated with generalized pollination systems (Waser et al. 1996; Aigner 2001).

A limitation of our study was that we were unable to observe flowers on the tops of the tall trees. However, we believe that this may not change the depicted pattern since *A. jamaiciencis* can forage on canopy trees (e.g., *Ficus* spp.) as well as on shrubs (e.g., *Piper* spp.) and herbs (e.g., *Solanum spp*) in the understory (García‐Estrada et al. 2012; Castro‐Luna and Galindo‐González 2012). Similarly, main diurnal visitors (Apidae bees), in addition to foraging on the understory, can forage in the top of the canopy (Ramalho 2004). Future research is needed to properly test this expectation by observing pollinators at different parts of the crown of *C. pentandra* .

In conclusion, the pollination system of *C. pentandra* in the Yucatan peninsula is highly generalized, where at least five groups of diurnal and nocturnal pollinators are involved. Diurnal and nocturnal pollinators contribute nearly equally to at least one quantity (visiting rate) and one quality pollination component (fruit and/or seed set). This indicates some redundance among different pollinator guilds. As expected, the contribution of diurnal and nocturnal pollinators to some quantity and quality components of pollination varied spatially and/or temporally. Therefore, this variability, along with the absence of specialized nectarivorous bats, is invoked as a probable ultimate factor promoting the observed generalization in the pollination system of the study species. Generalization in the pollination system and self‐compatibility exhibited by *C. pentandra* in the Yucatan Peninsula may be part of a strategy to deal with the uncertainty of having an opportunistic nocturnal pollinator.

## Author Contributions

**Henry Dzul‐Cauich:** conceptualization (lead), data curation (lead), formal analysis (lead), investigation (lead), methodology (lead), visualization (lead), writing – original draft (lead), writing – review and editing (supporting). **Kathryn E. Stoner:** conceptualization (equal), investigation (equal), methodology (equal), visualization (equal), writing – original draft (equal), writing – review and editing (lead). **Carlos N. Ibarra‐Cerdeña:** conceptualization (supporting), investigation (supporting), methodology (supporting), visualization (equal), writing – original draft (equal), writing – review and editing (equal). **Miguel A. Munguía‐Rosas:** conceptualization (lead), formal analysis (lead), investigation (lead), writing – original draft (lead), writing – review and editing (lead).

## Conflicts of Interest

The authors declare no conflicts of interest.

## Supporting information


Data S1.

## Data Availability

All raw data are available in the online Data S1.
